# Production of Protein Hydrolysates with Antioxidant and Antihypertensive Activity from Edible Larvae of *Aegiale hesperiaris* and *Comadia redtenbacheri*

**DOI:** 10.3390/foods14122124

**Published:** 2025-06-17

**Authors:** Eduardo R. Garrido-Ortiz, Jocksan I. Morales-Camacho

**Affiliations:** Department of Chemical, Food and Environmental Engineering, Universidad de las Américas Puebla, San Andrés Cholula 72810, Puebla, Mexico; eduardo.garridooz@udlap.mx

**Keywords:** edible insects, bioactive properties, antioxidant activity, angiotensin- converting enzyme (ACE) inhibitory activity, bioactive peptides, *Aegiale hesperiaris*, *Comadia redtenbacheri*, enzymatic hydrolysis

## Abstract

The search for sustainable and health-promoting food sources has increased interest in edible insects, which are rich in proteins and bioactive compounds with potential nutraceutical applications. In this study, we evaluated the bioactive properties of protein hydrolysates derived from *Aegiale hesperiaris* (maguey white worm, WW) and *Comadia redtenbacheri* (maguey red worm, RW), two culturally and economically significant insect species in Mexico. Hydrolysates were obtained via enzymatic treatments: either single hydrolysis with pepsin (PH) or sequential hydrolysis with pepsin followed by trypsin (PTH). The PTH hydrolysates exhibited strong antioxidant activity, with 2,2-azino-bis (3-ethylbenzothiazoline-6-sulfonic acid) (ABTS) radical inhibition above 90% and 2,2-diphenyl-1-picrylhydrazyl (DPPH) radical scavenging capacity between 75–85%. Additionally, they showed significant angiotensin-converting enzyme (ACE) inhibitory activity, reaching IC_50_ values of 0.35 and 0.017 μg/mL for WWPH and RWPH, respectively—the latter outperforming the commercial drug Enalapril (IC_50_ = 0.11 μg/mL). SDS-PAGE analysis revealed low molecular weight peptides (<10 kDa), especially between 5–9 kDa, associated with enhanced bioactivity. Peptides from RW also showed low Hill coefficients, suggesting a gradual and sustained interaction with ACE. These findings support the use of insect-derived hydrolysates as promising multifunctional ingredients for the development of functional foods targeting cardiovascular health.

## 1. Introduction

The consumption of insects as an alternative nutrient source has been a common practice in various cultures, benefiting from their high protein content and other essential components. In recent decades, the Food and Agriculture Organization of the United Nations (FAO) has recommended the inclusion of certain insect species in the human diet as a sustainable alternative to traditional animal protein sources, in order to meet growing nutritional demands and to reduce the environmental impact associated with conventional livestock production [1,2,3]. This has led to a new focus in food science research on the use of insects as food or feed ingredients. Currently, more than 2100 species of insects have been recorded as edible, with the Americas, Asia, and Africa being the regions with the highest number of identified species [4,5]. Among the edible species, Coleoptera (beetles) account for about 31%, followed by Lepidoptera (butterflies and moths) with 18%, Hymenoptera (ants and wasps) with 14%, and Orthoptera (crickets and grasshoppers) with about 13% [6].

From a nutritional point of view, insects are considered to be one of the best alternative sources of human food due to their high content of proteins, lipids, essential amino acids, fatty acids, dietary fiber, iron, zinc, vitamins such as B_12_, and, in some cases, folic acid [7,8,9]. On the other hand, the consumption of insects in different cultures has not been limited to their nutritional contribution, but some of their medicinal properties have also been identified. For centuries, various species of edible insects have been used for therapeutic purposes, a practice known as “entomotherapy” [10]. Recent studies have shown that edible insects provide bioactive compounds, because they present hormone or drug-like activity, with demonstrated antioxidant, anticancer, antithrombotic, antihypertensive, anti-obesity, anti-inflammatory, anti-diabetic, opioid, mineral-binding, immunomodulatory, anti-aging, and antimicrobial effects [11,12].

The most advanced studies in the production of bioactive compounds from edible insects have focused on the extraction of bioactive peptides, due to their high relevance to human health and their functional potential in food. These peptides are characterized by antioxidant, antihypertensive, antimicrobial, and other biological activities. They are generally obtained by processes such as digestion, fermentation, the use of commercial enzymes or chemical hydrolysis. Among these techniques, chemical hydrolysis has been shown to be the least suitable because it has limitations such as lack of specificity in protein hydrolysis, low yields in the production of functional peptides, and the generation of unwanted by-products that may affect the safety or functionality of the final product [13].

Enzymatic hydrolysis, on the other hand, has been shown to be an efficient method because it solubilizes some proteins, improves the selective activity on the substrate, and favors the release of bioactive compounds [14]. This method can use both endogenous and exogenous enzymes (alcalase, flavourzyme, corolase), obtaining protein hydrolysates with high specificity and minimal formation of by-products [15]. Therefore, enzymatic hydrolysis can modify the structure of proteins to enhance their nutraceutical properties without compromising their safety [16]. Recent studies have shown that the efficiency of enzymatic hydrolysis depends on several factors, including the enzyme-substrate ratio and reaction time. For example, in a study using *Bombyx mori* species, products obtained by simulated gastrointestinal digestion showed high antioxidant activity and the ability to inhibit angiotensin-converting enzyme (ACE), a valuable property for the treatment of hypertension [17,18,19]. Other reports with insects such as *Tenebrio molitor* (mealworm) and *Gryllus bimaculatus* (cricket) have shown that hydrolysis with digestive enzymes (such as pepsin, trypsin, and chymotrypsin) is particularly effective in generating peptides that inhibit key enzymes, such as α-glucosidase and ACE, suggesting possible applications for glucose and blood pressure control [11,20,21].

However, most studies to date have focused on a limited number of edible insect species, notably *Tenebrio molitor*, *Acheta domesticus*, and *Bombyx mori*, leaving a vast number of culturally important species, especially in countries like Mexico, largely unexplored. Despite its high diversity and long-standing tradition in entomophagy, where more than 549 species have been reported, Mexico has lagged behind in scientific research aimed at characterizing the bioactive potential of native edible insects. In contrast, European countries such as Italy, Belgium and the Netherlands, where insect consumption is limited, already count on well-developed regulatory frameworks and active research pipelines on insect-based ingredients [22]. In this context, two species with cultural and economic importance in Mexico *Aegiale hesperiaris* (white maguey worm, WW) and *Comadia redtenbacheri* (red maguey worm, RW) were selected for study. Both species are traditionally consumed in central Mexico, particularly in states such as Hidalgo, Puebla, and Oaxaca, where they are considered gastronomic delicacies and are often sold in local markets or featured in seasonal dishes [23]. These insects are part of the intangible heritage of local communities, yet their scientific potential remains understudied. While reports exist regarding their nutritional composition, no prior studies have evaluated their capacity to generate antioxidant or antihypertensive peptides through enzymatic hydrolysis. The growing commercial demand for wild edible insects, coupled with the absence of biosafety standards and the risk of overexploitation, underscores the urgency of developing scientific evidence that supports their sustainable use and functional value [24]. While antioxidant activity is a property widely reported in different natural sources, bioactive peptides offer distinct advantages such as low molecular weight, high bioavailability, and the potential for multifunctional effects, including enzyme inhibition relevant to metabolic health. Moreover, their derivation from underutilized and sustainable protein sources such as edible insects aligns with current priorities in functional food development. Therefore, evaluating the antioxidant and antihypertensive potential of peptides derived from *A. hesperiaris* and *C. redtenbacheri* is not only novel, but also strategically relevant to expanding the range of health-promoting ingredients available for nutraceutical applications. Therefore, the aim of this study was to evaluate their antioxidant and antihypertensive properties after being subjected to hydrolysis with pepsin alone (PH) or by sequential hydrolysis with pepsin followed by trypsin (PTH). This work contributes to filling the knowledge gap on native Mexican edible insects and supports their potential application in the development of functional foods and nutraceuticals.

## 2. Materials and Methods

### 2.1. Samples

Individuals of *Aegiale hesperiaris* (WW) and *Comadia redtenbacheri* (RW) species at the larval stage were collected directly from the leaves and stem of the maguey plant (*Agave salmiana*), in the Actopan region (20.287683, −98.912330), Hidalgo State, Mexico. For each species, a 1000 g sample of edible larvae was previously frozen (−20 °C) and subjected to lyophilization at −50 °C and 21.90 Pa using a laboratory-scale lyophilizer (Triad™ Labconco, Kansas City, MO, USA). The samples were then comminuted using a Nutribullet^®^ food processor (Los Angeles, CA, USA) at 10,000 rpm for 15 s to obtain a particle size < 0.15 mm. Both WW and RW flours were stored in airtight stand-up pouches at −20 °C until further use, in order to avoid any chemical, physical, or microbial degradation.

### 2.2. Chemical Reagents

2,2′-azino-bis(3-ethylbenzothiazoline-6-sulfonic acid) (ABTS), 2,2-diphenyl-1-picrylhydrazyl (DPPH), porcine gastric mucosal pepsin (≥ 250 U/mg solid; EC number 3.4.23.1), porcine pancreatic trypsin (1000–2000 BAEE U/mg solid; EC number 3.4.21.4), and a Precision Plus Protein™ Dual Color molecular weight marker were purchased from Bio-Rad Laboratories, Inc., Hercules, CA, USA. The ACE-WST kit was purchased from Dojindo Inc., Kumamoto, Japan.

### 2.3. Proximal Analysis

Moisture, nitrogen, lipid and ash contents were determined according to the official methods proposed by the AOAC [25]. Lipid content was determined using a Goldfish extractor (E-500 Büchi, Flawil, Switzerland) and hexane as solvent. Total nitrogen content was determined by the micro-Kjeldahl method, and in order to avoid overestimation of the protein content of WW and RW, a nitrogen to protein conversion factor of 4.76, proposed by Janssen et al. [26], was used. All determinations were carried out in triplicate (n=3).

### 2.4. Preparation of Protein Concentrates from Aegiale hesperiaris (WW) and Comadia redtenbacheri (RW)

WW and RW flours were defatted with 99% pure n-hexane according to the method of Kim et al. [27] with minor modifications. For each sample, 50 mL tubes were filled with a flour suspension containing 20% *w*/*v* solvent and mixed on an analogue roller stirrer (Cole-Parmer SRT6D, Fisher Scientific, Oxford, UK) at 27 rpm and 24 °C for 12 h. The fat-containing solvent was removed by gravity settling followed by careful decantation. This process was repeated 3 times. In a second step, proteins were isolated from defatted flours using the alkaline extraction method as described by Bose et al. [28] and Cortazar-Moya et al. [29] with modifications. In this step, the defatted powder (10% *w*/*v*) was suspended in denaturing buffer at (pH 7.2). The buffer composition was as follows: 20 mM Tris-HCl, 6 M urea and 0.2 M NaCl. The suspension was shaken on an analog roller mixer for 24 h at room temperature (22 °C); then, the mixture was centrifuged at 16,000 rpm (Hermle Z 326 K Centrifuge, Burladingen, Germany) for 20 min at 4 °C. The supernatant was then removed and the collected pellet was subjected to two further extractions using the same procedure. At the end of the procedure the pellet was isolated from the supernatant. For each meal, the supernatants obtained from the above extractions were pooled and dialyzed using a 43 mm × 27 mm cellulose membrane (Sigma-Aldrich, St. Louis, MO, USA), modifying the urea concentration to reduce it to zero. Thus, the supernatants were dialyzed in 800 mL of a solution (pH 7.5) containing 20 mM Tris-HCl, 3 M urea, and 0.2 M NaCl for 1 h at 24 °C. No vacuum or pressure was applied. This procedure was repeated for each urea concentration step (3 M to 0.25 M). Finally, the extracts were frozen, lyophilized and stored at −20 °C in the dark. The nitrogen content of these WW and RW protein concentrates (PC) was determined as described above, in triplicate (n=3) and the samples were coded as WWPC (WW protein concentrates) and RWPC (RW protein concentrates).

### 2.5. Enzymatic Hydrolysis

Protein hydrolysates were obtained by hydrolysis with pepsin alone (PH) and also by a sequential hydrolysis process with first pepsin and then trypsin (PTH). In both cases, the enzyme-substrate concentration was adjusted (1:10 E/S) according to that reported by Cortazar-Moya et al. [29]. For single hydrolysis (PH), 100 mg of WWPC and RWPC protein were hydrolyzed with pepsin at 37.5 °C and pH 2.0 in a citrate-phosphate buffer solution (0.1 M citric acid and 0.2 M sodium dibasic phosphate). The samples were incubated in a water bath with shaking at 80 rpm (Labtech, LSB-030S, Ortenberg, Germany) under dark conditions for 2 h. The pH was adjusted using 1 M HCl and monitored throughout the hydrolysis using a calibrated pH meter. Enzyme activity was then stopped by adjusting the pH to 7.0 using 1 M NaOH.

For sequential hydrolysis (PTH), the above procedure was followed with pepsin and upon completion, the pH was adjusted to 7.5 and trypsin was added as a secondary enzyme. Incubation was performed under the same conditions as for PH. Hydrolysis in the PTH procedure was stopped by adjusting the pH to 7.0 using 1 M HCl or NaOH as needed, and pH was monitored throughout. These hydrolysis conditions (37.5 °C, pH 2.0 for PH; pH 7.5 for PTH) were based on those previously reported by Cortazar-Moya et al. [29] for optimal release of bioactive peptides from insect proteins, and were not further optimized in this study. The hydrolysates obtained from the protein concentrates were cooled to room temperature (22 °C), centrifuged at 4 °C and fractionated using an Amicon^®^ Ultra ultrafiltration membrane (Millipore, Darmstadt, Germany) with a cut-off of 10 kDa. Filtration was performed by centrifugation at 1000 rpm and 4 °C, processing 4 mL of each sample for 20 min. The filtrates were collected and stored at −20 °C until further use. All hydrolysis reactions were performed in triplicate (n=3) for each treatment and insect species. For products obtained by PH and PTH, samples were coded as WWPH (WW hydrolyzed with pepsin alone), WWPTH (WW hydrolyzed with pepsin and trypsin), RWPH (RW hydrolyzed with pepsin alone) and RWPTH (RW hydrolyzed with pepsin and trypsin).

### 2.6. Sodium Dodecyl Sulfate-Polyacrylamide Gel Electrophoresis (SDS-PAGE)

SDS-PAGE electrophoresis of WW and RW protein concentrates, and their hydrolysates was performed according to the method described by Yi et al. [30] with minor modifications. For this analysis, the protein concentrates were diluted to a final concentration of 10 mg/mL in 20 mM Tris-HCl buffer, pH 8. WW and RW hydrolysates, both those obtained by simple hydrolysis (PH) and those obtained by sequential hydrolysis (PTH), were diluted in a 1:1 ratio in a solution of 20 mM Tris-HCl buffer, pH 8. For reducing conditions, each sample was mixed 1:1 with a loading buffer consisting of Tris-HCl (20 mM and pH 8), 5% (*w*/*v*) SDS, 0.016% (*w*/*v*) 2-mercaptoethanol and 0.02% (*w*/*v*) bromophenol blue. A 6% stacking gel and a 12% separating gel were used for protein separation. A protein marker (catalog no. 266632, Bio-Rad) was used as a molecular weight standard. The gels were stained with Coomassie blue R250 (Bio-Rad) and destained with a 10% methanol and 7% acetic acid solution according to Laemmli’s protocol [31].

### 2.7. Determination of Antioxidant Capacity

#### 2.7.1. DPPH Radical Scavenging Activity

The DPPH assay was performed according to the method of Brand-Williams et al. [32] with slight modifications. Each activity calculation was expressed as the mean and standard deviation of three replicates. A 20 μL volume of sample was mixed with 200 μL of a 0.1 mM DPPH solution in 80% ethanol. The mixture was vortexed and incubated in the dark at 22 °C for 30 min, and the absorbance of the mixture was measured at 517 nm using a UV-Vis Multiskan Sky microplate spectrophotometer (Thermo Scientific, Singapore). The activity was expressed as the percentage inhibition of DPPH radical relative to the control, using Equation (1). A calibration curve with Trolox (0–100 ppm) was used to validate the linear response of the assay system, although antioxidant activity was expressed as percentage inhibition and not in Trolox equivalents. All measurements were performed in triplicate (n=3), using deionized water as a blank. The method showed good linearity ($R^{2}>0.95$).

(1) $$\%\mathrm{DPPH}-\mathrm{RS}=\frac{A_{0}\left(517\;\mathrm{nm}\right)-A_{\mathrm{sample}}\left(517\;\mathrm{nm}\right)}{A_{0}\left(517\;\mathrm{nm}\right)}\times 100$$

where $A_{\mathrm{sample}}\left(517\;\mathrm{nm}\right)$ is the absorbance of the sample and $A_{0}\left(517\;\mathrm{nm}\right)$ is the absorbance of the control.

The IC_50_ was determined by evaluating the free radical scavenging activity of sequential dilutions (0.025–100 mg/mL) of each sample and interpolating the peptide concentration at which the percentage inhibition reached 50%. The IC_50_ value was calculated for all samples: WWPC, RWPC, and filtered samples obtained by PH and PTH hydrolysis.

#### 2.7.2. ABTS Cation Radical Scavenging Activity

The activity to inhibit the ABTS radical was determined according to Re et al. [33] with some modifications. The ABTS radical solution was prepared using a solution of 2.45 mM potassium persulfate and 2,2′-azino-bis(3-ethylbenzothiazoline-6-sulfonic acid) 7 mM in 10 mL Milli-Q ultrapure water. The solution was incubated in the dark at 22 °C for 16 h and then diluted with 80% ethanol to an absorbance of 0.7 ± 0.2 at 754 nm. The assay was performed by adding 200 μL of ABTS radical solution to 20 μL of sample. The mixture was kept at room temperature for 6 min in the dark before measuring the absorbance at 754 nm in a UV-Vis Multiskan Sky UV-Vis microplate spectrophotometer (Thermo Scientific, Singapore). Deionized water was used as a blank. The inhibition percentage was calculated according to Equation (2).

(2) $$\%\mathrm{ABTS}-\mathrm{RS}=\frac{A_{\mathrm{control}}\left(754\;\mathrm{nm}\right)-A_{\mathrm{sample}}\left(754\;\mathrm{nm}\right)}{A_{\mathrm{control}}\left(754\;\mathrm{nm}\right)}\times 100$$

where $A_{\mathrm{sample}}\left(754\;\mathrm{nm}\right)$ is the absorbance of the ABTS solution with the sample; and $A_{\mathrm{control}}\left(754\;\mathrm{nm}\right)$ is the absorbance of the ABTS solution without the sample.

A calibration curve with Trolox (0–100 ppm) was used to validate the linear response of the assay system, although antioxidant activity was expressed as percentage inhibition and not in Trolox equivalents. All measurements were performed in triplicate (n=3), using deionized water as a blank. The method showed good linearity ($R^{2}>0.97$). The results were calculated as the IC_50_ (inhibitory concentration) value and determined by evaluating the free radical scavenging activity of different dilutions (0.025–100 mg/mL) of each sample and interpolating the peptide concentration at which the percentage inhibition reached 50%. The IC_50_ value was calculated for all the samples: WWPC, RWPC and for the hydrolyzed samples (WWPH, WWPTH, RWPH, and RWPTH).

### 2.8. Determination of Percent ACE Inhibition

Angiotensin-converting enzyme (ACE) inhibition of the obtained samples (WWPC, RWPC, WWPH, WWPTH, RWPH and RWPTH) was determined according to the colorimetric method described by Anuduang et al. [34] and Iwamoto et al. [35] with some modifications (Table 1).

An ACE-WST kit (Dojindo Inc., Kumamoto, Japan), a UV-Vis Multiskan Sky microplate UV-Vis spectrophotometer (Thermo Scientific, Singapore) and Enalapril (Ultra Lgen^®^, London, UK; 10 mg) as a commercial ACE inhibitor (positive control) were used for the assay. On the other hand, samples containing 100 mg/mL of protein (WWPC and RWPC) or their hydrolysates by PH and PTH were diluted to obtain concentrations ranging from 0.1 mg/mL to 100 mg/mL. First, 10 μL of sample, Enalapril or distilled water (used for the positive control without inhibition (B_1_) and the reagent blank (B_2_)) were added to each well in a 96-well plate. Then 10 μL of substrate buffer was added to each well, followed by 10 μL of enzyme working solution to each sample and control well. The plate was incubated at 37 °C for 1 h. Then, 100 μL of indicator working solution was added to each well containing the reagent solutions, followed by incubation at 37 °C for 10 min. Finally, the absorbance of the samples, B_1_ and B_2_ was measured at 450 nm, and all assays were performed in triplicate. To calculate the percentage inhibition of the samples subjected to the assay, Equation (3) was used.

(3) $$\%\mathrm{ACE}\;\mathrm{inhibition}=\frac{A_{B_{1}}-A_{\mathrm{sample}}}{A_{B_{1}}-A_{B_{2}}}\times 100$$

where $A_{B_{1}}$ is the absorbance of the positive control (without inhibition), $A_{\mathrm{sample}}$ is the absorbance of the sample and $A_{B_{2}}$ is the absorbance of the reagent blank.

The IC_50_ was determined by evaluating the ACE inhibition activity of different dilutions of each sample. The logistic dose-response model was used to determine the IC_50_ value. The experimental data were fitted with a sigmoid curve, modeling the percentage inhibition as a function of inhibitor concentration. The IC_50_ value, which corresponds to the concentration at which 50% inhibition is achieved, was calculated by fitting the Hill equation to the dose-response curve and determining the coefficient “*n*” from the experimental data according to Equation (4). The IC_50_ value was calculated for all samples (WWPC, RWPC, WWPH, WWPTH, RWPH and RWPTH). All measurements were performed in duplicate (n=2), and IC_50_ values were determined using nonlinear regression based on a four-parameter logistic model.

(4) $$Y=\frac{Y_{\max}}{1+{\left(\frac{{\mathrm{IC}}_{50}}{X}\right)}^{n}}$$

where $Y_{\max}$ is the maximum effect, ${\mathrm{IC}}_{50}$ is the inhibitor concentration at which 50% of the maximum effect is reached, *X* is the inhibitor concentration and “*n*” is the slope (Hill’s coefficient). The IC50 value was calculated for all samples (WWPC, RWPC and the filtered samples obtained from the PH and PTH treatments).

### 2.9. Statistical Analysis

All experimental data are presented as mean ± standard deviation. The dependent variables were antioxidant activity (expressed as percentage inhibition), ACE inhibition percentage, and IC_50_ values. Minitab^®^ 18 software (Minitab Inc., State College, PA, USA) was used to perform one-way analysis of variance (ANOVA) to evaluate differences among treatments. When significant differences were found (p<0.05), Tukey’s multiple comparison test was applied. All analyses were conducted based on three replicates per treatment (n=3).

## 3. Results and Discussion

### 3.1. Nutritional Composition of WW and RW Flours

The composition of WW (*A. hesperiaris*) and RW (*C. redtenbacheri*) flours was determined by proximate analysis, with the aim of determining crude protein, lipid, carbohydrate, fiber, ash content, nitrogen-free extract (NFE) and caloric content; the results are presented in Table 2.

The proximate composition results showed a protein content of 28.11% ± 0.40 and 22.87% ± 0.27 for WW and RW flours, respectively. When compared with conventional protein sources, such as soy (36–56%) or casein (around 38%), these values are lower. However, considering that these correspond to whole insect flours (including chitin and lipids), they remain within an acceptable range for biotechnological applications.

These values may appear lower compared to previous studies using the standard nitrogen to protein conversion factor (35–65% on a dry basis) [36]. However, this factor (6.25), widely used in traditional feeds, has been shown to be inadequate for edible insects due to the presence of non-protein nitrogen (NPN) in compounds such as chitin, nucleotides, and excretions present in their digestive system [26]. This leads to an overestimation of protein content by up to 20%, especially in species with a prominent exoskeleton, such as insects. To improve accuracy, an adjusted conversion factor (4.67) based on amino acid analysis was used, which more realistically reflects the actual protein content in edible insects and has been widely accepted for compositional studies. This approach reduces overestimation and places the true protein content of insects in a range comparable to that of other alternative food sources.

Notably, a protein content above 20% is considered adequate for enzymatic hydrolysis aimed at generating peptides with biological activity, such as antioxidant and antihypertensive effects. Despite this correction, the values obtained in this study for both insect species exceed the 20% threshold and are considered suitable for biotechnological applications such as enzymatic hydrolysis, where a protein content higher than 20% allows the extraction of bioactive peptides with functional properties such as solubility and antioxidant capacity [37,38].

Regarding the lipid content, it was observed that WW had a moderate level of 32.63% ± 0.20, while RW had a significantly higher value (p<0.05), reaching 56.22% ± 1.50. This aligns with previous studies reporting that Lepidoptera larvae tend to accumulate substantial lipid reserves, often surpassing 40%. Such high lipid content may influence the solubility and extraction of proteins, as it is known that lipid removal can increase the purity of isolated proteins by up to 50%, potentially improving the yield and functional activity of protein hydrolysates. For example, protein purities of 78.5% have been reported in *Acheta domesticus* and *Tenebrio molitor* after lipid removal [37,38,39].

Finally, it is important to consider that differences in proximal composition may be due both to intrinsic species variation and processing conditions [40,41]. However, the values obtained in this study are within the ranges reported for edible insects and, most importantly, provide a compositional basis that supports their application in bioactive peptide production, as demonstrated in later sections.

### 3.2. WW and RW Concentrates and Their Protein Profile

Protein concentrates subjected to alkaline hydrolysis showed a significant increase in protein content after the concentration process for both edible insect species. In WW, the protein content increased from 28.07% to 61.01% ± 2.14, while in RW the change was from 22.84% to 45.73% ± 3.92. This is more than double the initial protein concentration in both cases. This increase is due to the protein concentration process, which reduces the content of other components, such as lipids and carbohydrates, thus increasing the relative proportion of protein [30]. The improvement in protein content suggests that the concentrates obtained could be an important source of protein for the development of nutraceutical products and functional foods [42].

**Figure 1:**
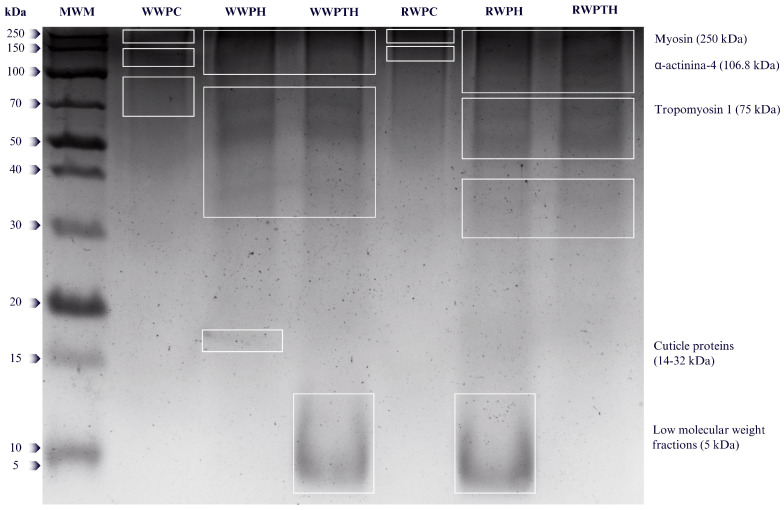
SDS-PAGE patterns of *Aegiale hesperiaris* and *Comadia redtenbacheri* concentrates and hydrolysates. MWM: molecular weight marker; WWPC: *Aegiale hesperiaris* protein concentrate; WWPH: single hydrolysis (pepsin) of *Aegiale hesperiaris* extracts; WWPTH: sequential hydrolysis (pepsin-trypsin) of *Aegiale hesperiaris* extracts; RWPC: *Comadia redtenbacheri* protein concentrate; RWPH: single hydrolysis (pepsin) of *Comadia redtenbacheri* extracts; RWPTH: sequential hydrolysis (pepsin-trypsin) of *Comadia redtenbacheri* extracts.

Protein characterization using the SDS-PAGE technique allowed the identification of the profile of proteins present in the concentrates and hydrolysates, especially those dependent on disulfide bonds. Figure 1 shows the molecular weight pattern of the protein concentrates (PC) and the WW and RW hydrolysates.

Several protein species were identified in the edible insect extracts, with well-defined bands corresponding to structural proteins reported in other studies. The major proteins identified include myosin (250 kDa), α-actinin-4 (107 kDa), tropomyosin 1 (75 kDa), and cuticle proteins (14–32 kDa), which play a key role in muscle structure and are characteristic of many organisms, including insects such as *Bombyx mori* and other edible species [17,43,44,45]. In the study by Yi et al. [46], in vitro digestion of *Tenebrio molitor* protein fractions was performed using pepsin and pancreatin to simulate gastric and duodenal conditions. In SDS-PAGE analyses, muscle proteins, such as actin (30–50 kDa) and tropomyosin (∼32 kDa), were found to be more resistant to enzymatic digestion, suggesting that the structural matrix influences enzyme accessibility and amino acid release during digestion.

In this study, intense bands were observed in untreated concentrates (WWPC and RWPC) in the 75–250 kDa region, which match the size range of structural proteins such as myosin, tropomyosin, and α-actinin. After single (PH) and sequential (PTH) enzymatic hydrolysis, a significant degradation of these proteins was observed. For WWPH and RWPH, new bands appeared in the 14–32 kDa range, consistent with partial breakdown of cuticle-associated proteins. The most pronounced degradation was seen in WWPTH and RWPTH, which exhibited diffuse bands near 5 kDa, particularly in RWPTH. This treatment also showed the highest ACE inhibitory activity, suggesting that peptides in the <10 kDa range may be responsible for the enhanced bioactivity.Although densitometric quantification was not performed, the differential intensity and distribution of bands between treatments clearly demonstrate the impact of enzymatic processing on the protein profile of both species.

This is consistent with the results obtained after single and sequential hydrolysis of RW and WW where the range of protein molecular weights observed was much broader, with low molecular weight bands in the visible range of 5–70 kDa (Figure 1). SDS-PAGE analysis under reducing conditions, which facilitates the cleavage of disulfide bonds, allowed us to observe these proteins individually, highlighting the functional diversity of edible insect proteins. These proteins are not only essential for insect survival and adaptation, but also of great interest for biotechnological and nutraceutical applications due to their unique structural and functional properties [47].

### 3.3. Antioxidant Properties of Protein Concentrates and Hydrolysates

The antioxidant activity of protein concentrates and hydrolysates was evaluated using ABTS and DPPH assays.

**Figure 2:**
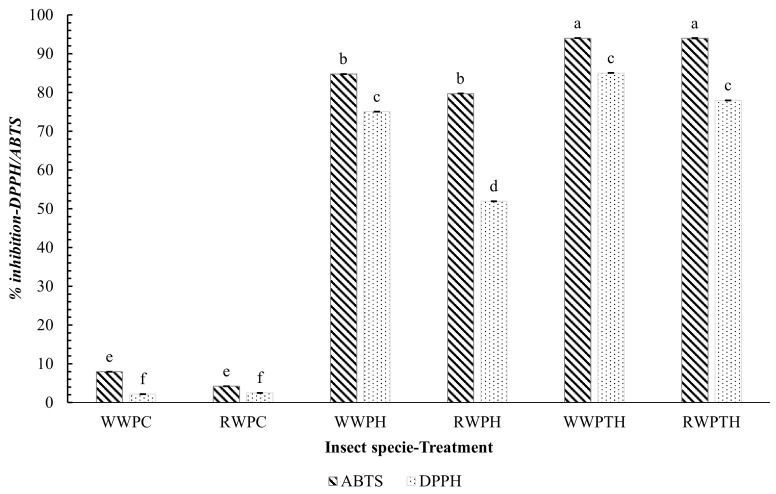
Antioxidant activity of protein concentrates and hydrolysates of *Aegiale hesperiaris* (WW) and *Comadia redtenbacheri* (RW). WWPC: *Aegiale hesperiaris* protein concentrate; WWPH: single hydrolysis (pepsin) of *Aegiale hesperiaris* extracts; WWPTH: sequential hydrolysis (pepsin-trypsin) of *Aegiale hesperiaris* extracts; RWPC: *Comadia redtenbacheri* protein concentrate; RWPH: single hydrolysis (pepsin) of *Comadia redtenbacheri* extracts; RWPTH: sequential hydrolysis (pepsin-trypsin) of *Comadia redtenbacheri* extracts. Different letters (a–f) between samples indicate significant differences (*p* < 0.05). Results are shown as mean ± standard deviation (*n* = 3).

As shown in Figure 2, the non-hydrolyzed samples (WWPC and RWPC) exhibited low inhibition percentages (<10%), confirming that enzymatic hydrolysis is essential to enhance antioxidant capacity. WWPH showed the highest antioxidant activity, reaching 94% ± 0.004 inhibition in ABTS and 84.78% ± 0.003 in DPPH, followed by RWPH, which showed moderate activity (ABTS: 79.72% ± 0.010, DPPH: 51.95% ± 0.030). Sequential hydrolysis (WWPTH and RWPTH) further improved radical scavenging capacity, with inhibition values exceeding 90% in ABTS and ranging from 75–85% in DPPH. These values were significantly higher than those of single hydrolysates and protein concentrates (p<0.05), highlighting the enhanced efficiency of sequential enzymatic treatment. IC_50_ values supported these trends, as shown in Table 3. In the DPPH assay, WWPH and RWPH showed values of 64.68 ± 5.33 and 81.01 ± 4.28 mg/mL, respectively, while WWPTH and RWPTH showed 51.16 ± 3.56 and 62.57 ± 5.12 mg/mL. Similarly, in the ABTS assay, WWPH and RWPH exhibited IC_50_ values of 46.31 ± 5.81 and 39.87 ± 6.43 mg/mL, whereas WWPTH and RWPTH showed significantly lower IC_50_ values of 38.31 ± 6.18 and 19.86 ± 2.81 mg/mL, respectively.

Although these values were not statistically compared, IC_50_ values below 100 mg/mL are generally interpreted as indicative of moderate to high antioxidant potential in peptide-rich systems [48,49,50]. The antioxidant capacity observed in sequential hydrolysates may be associated with the release of low-molecular-weight peptides (<10 kDa), which are more efficient in radical scavenging. This effect is often attributed to the presence of hydrophobic amino acids such as Tyr, Phe, and Pro, which enhance electron or hydrogen donation to neutralize reactive species [51,52]. These findings are consistent with previous studies on insects such as *Alphitobius diaperinus*, where ABTS radical inhibition above 90% was reported in peptide fractions [15], and in *Gryllus assimilis*, whose hydrolysates obtained with alcalase and neutrase showed a 160% increase in total antioxidant activity [21]. Similarly, hydrolysates of *Bombyx mori* prepared with Flavourzyme showed significant ROS reduction in HepG2 cells [53].

While other insect species have shown similar antioxidant trends, this study demonstrates for the first time that hydrolysates from *Aegiale hesperiaris* and *Comadia redtenbacheri* exhibit antioxidant profiles comparable to widely studied edible insects. These results highlight the potential of sequential hydrolysis as an effective strategy to obtain antioxidant peptides from underutilized insect sources, contributing to the development of sustainable functional ingredients.

### 3.4. Potential of Protein Concentrates and Hydrolysates to Inhibit ACE

**Figure 3:**
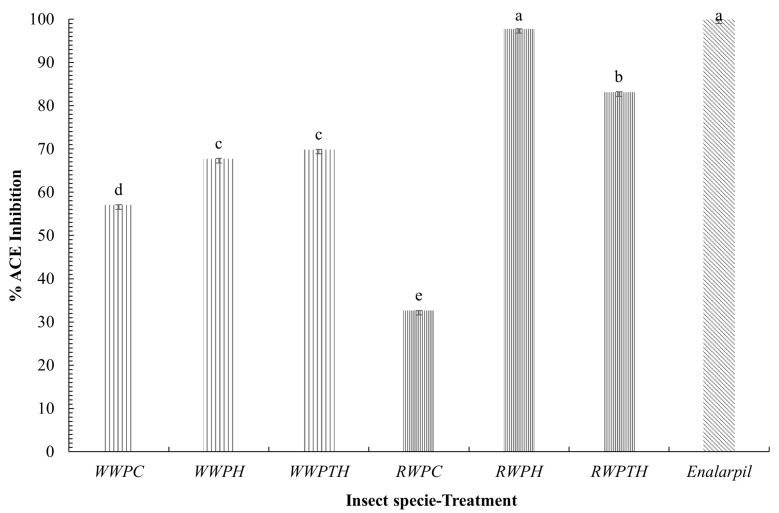
ACE inhibition of protein concentrates and hydrolysates from *Aegiale hesperiaris* and *Comadia redtenbacheri*. Values are expressed as mean ± SD of duplicate assays. The statistical difference (*p* < 0.05) with respect to the two species in their different treatments and the commercial inhibitor (Enalapril, control +) is presented with the letters a–e.

Figure 3 shows the percentage of ACE inhibition for protein concentrates and their hydrolysates from both WW and RW, compared with Enalapril as the positive control (10 µg/mL). In WW samples, enzymatic hydrolysis improved the inhibitory activity: WWPC showed 57.03% ± 0.057 inhibition, while WWPH and WWPTH reached 67.73% ± 0.065 and 69.84% ± 0.009, respectively. However, no significant differences were found between PH and PTH hydrolysates (p>0.05). In contrast, RW samples showed a stronger response. RWPC had the lowest inhibition (32.63% ± 0.018), while RWPH and RWPTH showed significant increases to 97.75% ± 0.018 and 83.15% ± 0.058, respectively. Notably, RWPH achieved inhibition statistically similar to Enalapril (99.98% ± 0.003) (p>0.05), suggesting that peptides generated from RW by single hydrolysis may exhibit comparable ACE inhibitory activity to the reference drug.

The results indicate that RW has greater potential than WW to release ACE inhibitory peptides, particularly after single hydrolysis. The differences observed may relate to species-specific factors such as initial protein composition, peptide release efficiency, or enzyme accessibility to cleavage sites [54,55]. Enzymatic hydrolysis is a commonly used method for releasing bioactive peptides from proteins because it allows precise control over the degree of hydrolysis and the specificity of the cleavage sites [56,57]. However, the results are dependent on the processing conditions, enzymes and molecular weight of the isolated peptides. alcalase, neutratase, chymotrypsin, pepsin and trypsin are the most commonly used enzymes for protein hydrolysis because they generate different peptide profiles due to their specificity for certain peptide bonds. The high ACE inhibition observed in RWPH hydrolysates compared to protein concentrates and sequential hydrolysates suggests that this process produces peptides with higher bioactive activity. This may be due to the release of specific peptides in this process that have a higher affinity for ACE, and effectively act as positive inhibitors [58,59,60]. On the other hand, in enzyme inhibition studies, it is essential to analyze various parameters that describe the interaction between an inhibitor and its target enzyme. In addition to assessing the potency of the inhibitor, these parameters provide detailed information about the molecular dynamics of the inhibition process [61,62]. Among the most commonly used is the IC_50_, which represents the concentration of the inhibitor required to reduce enzyme activity by 50%, providing a direct measure of inhibitor potency. Another relevant parameter is the Hill coefficient (*n*), which is obtained by fitting the experimental data to the Hill equation. This coefficient allows the evaluation of the cooperativity in the binding of the inhibitor to the enzyme: an n>1 indicates positive cooperativity, whereas an n<1 indicates negative or null cooperativity [63]. However, in both cases, inhibition of the enzyme occurs. To further evaluate inhibitory potential, IC_50_ values and Hill coefficients (*n*) were calculated using the logistic dose-response model.

**Figure 4:**
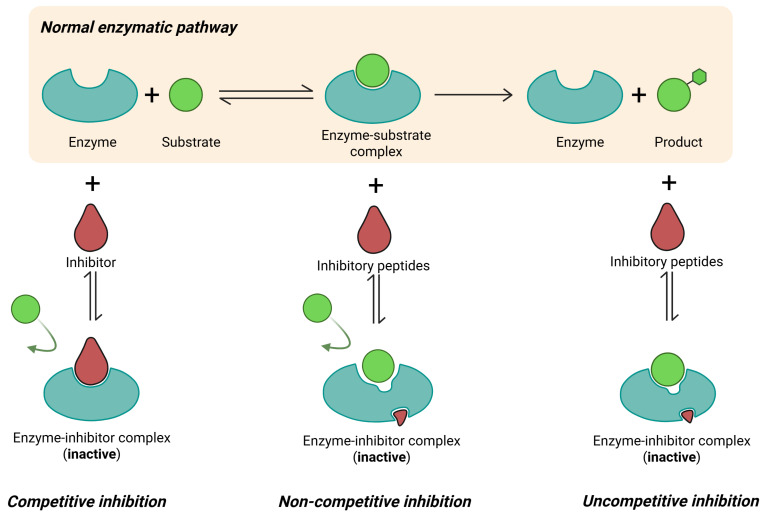
Mechanisms of enzyme inhibition comparing the action of the commercial inhibitor (Enalapril) and the hydrolysates of *Aegiale hesperiaris* and *Comadia redtenbacheri* on ACE, according to the Hill coefficient “*n*” and the type of inhibition.

Table 4 summarizes these parameters. In WW samples, the IC_50_ decreased from 0.73 µg/mL ± 0.047 (WWPC) to 0.58 µg/mL ± 0.018 (WWPH) and 0.35 µg/mL ± 0.04 (WWPTH), showing a modest improvement. The Hill coefficient remained low (n=0.13–0.40), indicating low cooperativity. In RW, hydrolysis caused a dramatic reduction in IC_50_: from 961.96 µg/mL ± 0.005 in RWPC to 0.061 µg/mL ± 0.009 in RWPH and 0.017 µg/mL ± 0.002 in RWPTH. The RWPTH sample showed even stronger inhibition than Enalapril (IC_50_ = 0.11 µg/mL ± 0.010), while RWPH showed a statistically similar value. These findings confirm that sequential hydrolysis with pepsin and trypsin releases highly potent inhibitory peptides in RW. When comparing the treatments, it was observed that WWPTH (0.35 µg/mL) and RWPTH (0.017 µg/mL) had the lowest IC_50_ values, indicating that sequential hydrolysis produces peptides with higher ACE inhibitory capacity in both insect species. However, the improvement was much more pronounced in RW, where the IC_50_ decreased by more than 50,000-fold between RWPC and RWPH, in contrast to WW where the decrease was approximately 2-fold between WWPC and WWPTH. The fit of Hill’s logistic model for IC_50_ determination was good in all cases, with *R*^2^ values above 0.90, indicating that the experimental data fit Hill’s equation well. Overall, the results reflect that sequential hydrolysis (PTH) was the most effective treatment to enhance ACE inhibition in both insect species, especially in RW, reaching values up to 0.99, indicating that this model describes the dose-response relationship of these inhibitors with high accuracy [64]. Figure 4 illustrates the inhibitory mechanism of ACE in the presence and absence of inhibitors, using Enalapril and the hydrolysates from RW and WW as functional models. In the absence of any inhibitor, ACE remains fully active and capable of converting angiotensin I into angiotensin II, promoting vasoconstriction and increased blood pressure. When Enalapril is present, it binds directly to the ACE active site with high specificity and affinity, producing near-total inhibition even at low concentrations. This is reflected in its steep dose-response curve and high Hill coefficient, which indicate strong cooperativity and rapid inhibition [62,65]. Hydrolysates from *Comadia redtenbacheri* (RW), particularly RWPTH, contain peptides that appear to mimic this inhibitory effect, binding to ACE and effectively blocking its function. However, the interaction is less cooperative, as shown by the lower Hill coefficient (n=0.12–0.48), meaning the inhibition increases more gradually with concentration. This behavior can be advantageous in nutraceutical applications where controlled, dose-dependent modulation of blood pressure is preferred over abrupt responses. In contrast, hydrolysates from *Aegiale hesperiaris* (WW) also inhibit ACE, but less effectively. The peptides generated may lack specific structural motifs that promote strong enzyme binding, or their release efficiency may be lower, resulting in moderate inhibition and a flatter dose–response curve.

The Hill coefficient (*n*) is particularly relevant in functional formulation, as it reflects how inhibition progresses with concentration. Low *n* values indicate gradual responses, which are useful in food-based interventions where stability and predictability of effect are important. In contrast, high *n* values, such as in Enalapril (n=0.90), correspond to sharp inhibition profiles, more typical of pharmaceutical agents [66]. While comparisons to pharmaceutical inhibitors must be interpreted cautiously, the IC_50_ of RWPTH (0.017 µg/mL) was lower than that of Enalapril, indicating strong in vitro inhibition. However, ACE inhibition assays do not reflect peptide digestion, absorption, or bioavailability, which limits clinical extrapolation. Thus, the activity of RW hydrolysates should be viewed as complementary to pharmacological strategies, not as a replacement. Antihypertensive peptides exhibit remarkable affinity for ACE binding due to their C-terminal amino acid sequences. Previous studies have identified a wide variety of bioactive peptides with antihypertensive activity in edible insects. In *B. mori*, a peptide with the sequence Ala-Ser- was identified, with an IC_50_ of 102.15 µM, indicating that its ACE inhibitory activity is influenced by the presence of hydrophobic residues in its structure. The same study reported that silkworm protein hydrolysates showed significant inhibition, with IC_50_ values of 596.11 µg/mL at ultrafiltration stages (<5 kDa), indicating that, although less potent than other insects, they can be optimized by fractionation and purification [67]. In *T. molitor*, the Tyr-Ala-Asn peptide showed an IC_50_ of 17 µg/mL, indicating a strong ability to inhibit ACE. Moreover, in the same study, it was observed that low molecular weight fractions (180–500 Da) had higher antihypertensive activity, suggesting that small peptides may have better bioavailability and efficacy [68]. In *Schistocerca gregaria*, peptides with KVEGDLK, YETGNGIK and AIGVGAIR sequences showed an IC_50_ of 3.95 µg/mL, highlighting their high ACE inhibitory capacity, suggesting that Orthoptera are a promising source of bioactive compounds [69]. Similarly, in *Spodoptera littoralis*, protein hydrolysates showed an IC_50_ of 320 µg/mL after gastrointestinal digestion, and 211 µg/mL after hydrolysis with mucosal enzymes, indicating that the type of digestion affects the inhibitory activity [70]. In another study, peptides (<3 kDa) obtained from *Alphitobius diaperinus* were found to have IC_50_ values (111.33 ± 21.3 µg/mL) comparable to those of milk- and fish-derived peptides, suggesting their potential application in functional foods [71]. Furthermore, an analysis of *Acheta domesticus* and *Gryllodes sigillatus* showed that hydrolysates with pepsin and alcalase significantly reduced ACE activity, with IC_50_ values between 5 and 20 µg/mL, highlighting the efficiency of these treatments in generating bioactive peptides [72].

Altogether, these results highlight the potential of *Comadia redtenbacheri* as a promising source of bioactive peptides with antihypertensive activity. Sequential enzymatic hydrolysis proved to be a highly effective strategy, positioning this species as a viable ingredient in functional food development [73,74]. Nonetheless, the observed inhibition suggests that RW-derived hydrolysates may serve as complementary dietary strategies in the management of blood pressure, particularly in populations seeking food-based approaches to cardiovascular health. Further studies including simulated digestion, Caco-2 transport, and in vivo models would help clarify their real efficacy and bioavailability.

## 4. Conclusions

This study demonstrated that enzymatic hydrolysates derived from *Aegiale hesperiaris* (WW) and *Comadia redtenbacheri* (RW) possess significant antioxidant and ACE inhibitory activity, particularly after sequential hydrolysis with pepsin and trypsin. Among the two species, the hydrolysates of RW, especially RWPTH exhibited the most potent bioactivity, with IC_50_ values and inhibition profiles comparable to or even exceeding those of the pharmaceutical reference Enalapril in vitro. However, while these findings are promising, they must be interpreted within the constraints of the experimental model. The assays used to inhibit DPPH, ABTS, and ACE are in vitro approximations that do not fully capture the complexity of digestion, absorption, peptide metabolism, and systemic bioavailability in vivo. Furthermore, variability in insect composition due to environmental, developmental, or seasonal factors can influence peptide yield and functionality, which must be considered when scaling up for industrial or nutritional use.

Specific applications may include their use as active components in functional beverages, protein-enriched snacks, or powdered dietary supplements targeting hypertension and oxidative stress. Furthermore, their gradual inhibitory profile reflected in low Hill coefficients suggests that they may offer a safer alternative to abrupt-acting pharmacological inhibitors in functional food contexts.

Future research should focus on confirming the bioactivity of these peptides through simulated gastrointestinal digestion models, cell-based transport and uptake studies (e.g., using Caco-2 cells) and in vivo trials in hypertensive models. Additionally, identifying the specific peptide sequences responsible for the observed effects using LC–MS/MS and exploring formulation stability within real food systems will be essential to advance from laboratory findings to commercial applications. Lastly, this study provides the first evidence that hydrolysates from culturally significant edible insects in Mexico can serve as novel sources of bioactive peptides. Beyond their traditional culinary value, these species offer a scientifically supported opportunity to contribute to the development of next-generation functional foods.

## Figures and Tables

**Table 1:** ACE inhibition assay (amount of sample and reagent used per well).

Reactant	Sample (µL)	B_1_ (µL)	B_2_ (µL)
Sample solution	10	-	-
Deionized water	-	10	20
Substrate buffer	10	10	10
Enzyme working solution	10	10	-
Sample incubation at 37 °C × 1 h			
Working solution indicator	100	100	100
Sample incubation at 37 °C × 10 min			

B_1_: positive control with no inhibition and B_2_: reagent blank.

**Table 2:** Proximal composition of WW and RW flours (% dry basis).

Flour Sample	%Protein **	%Fat	%Ash	%Crude fiber	NFE * (%)
*A. hesperiaris* (WW)	28.11 ± 0.40 ^a^	32.63 ± 0.20 ^b^	2.25 ± 0.70 ^b^	9.60 ± 0.07 ^b^	27.41 ^a^
*C. redtenbacheri* (RW)	22.87 ± 0.27 ^b^	56.22 ± 1.50 ^a^	2.08 ± 0.36 ^b^	10.53 ± 0.19 ^a^	8.30 ^c^

* Nitrogen free extract [100 − (protein + crude fat + ash + crude fiber)]. ** Protein conversion factor = 4.76 according to Janssen et al. [26]. Values with different lowercase letters within the same row are significantly different (p≤0.05).

**Table 3:** Antioxidant activity of *A. hesperiaris* and *C. redtenbacheri* hydrolysates.

Insect Species	Sample	DPPH Radical Scavenging Activity (IC_50_ mg/mL)	ABTS Radical Scavenging Activity (IC_50_ mg/mL)
	WWPC	NC	NC
*A. hesperiaris* (WW)	WWPH	64.68 ± 5.33 ^a^	46.31 ± 5.81 ^b^
	WWPTH	51.16 ± 3.56 ^a^	38.31 ± 6.18 ^b^
	RWPC	NC	NC
*C. retdenbacheri* (RW)	RWPH	81.01 ± 4.28 ^a^	39.87 ± 6.43 ^b^
	RWPTH	62.57 ± 5.12 ^a^	19.86 ± 2.81 ^b^

NC: not calculable, WWPC: *Aegiale hesperiaris* protein concentrate, WWPH: single hydrolysis with pepsin, of *Aegiale hesperiaris* extracts and WWPTH: sequential hydrolysis (pepsin-trypsin) of *Aegiale hesperiaris* extracts; RWPC: *Comadia redtenbacheri* protein concentrate, RWPH: single hydrolysis (pepsin) of *Comadia redtenbacheri* extracts and RWPTH: sequential hydrolysis (pepsin-trypsin) of *Comadia redtenbacheri* extracts. Values are expressed as mean ± standard deviation of triplicate assays. Different letters in each row represent significant difference (*p* < 0.05) in their different treatments.

**Table 4:** ACE inhibitory capacity of hydrolysates obtained from *A. hesperiaris* and *C. redtenbacheri*.

Insect Species	Treatment	ACE Inhibitory Activity (IC_50_ µg/mL)	Hill’s Coefficient (*n*)	Correlation Coefficient ($R^{2}$)
	WWPC	0.73 ± 0.047 ^A^	0.13	0.90
*A. hesperiaris* (WW)	WWPH	0.58 ± 0.018 ^B^	0.40	0.96
	WWPTH	0.35 ± 0.004 ^C^	0.39	0.95
	RWPC	961.96 ± 0.005 ^a^	0.12	0.95
*C. retdenbacheri* (RW)	RWPH	0.061 ± 0.009 ^c^	0.48	0.99
	RWPTH	0.017 ± 0.002 ^d^	0.28	0.98
Enalapril *	NT	0.11 ± 0.028 ^D,b^	0.90	0.99

WWPC: protein concentrate of *Aegiale hesperiaris*, WWPH: single hydrolysis (pepsin) of *Aegiale hesperiaris* extracts and WWPTH: sequential hydrolysis (pepsin-trypsin) of *Aegiale hesperiaris* extracts; RWPC: *Comadia redtenbacheri* protein concentrate, RWPH: single hydrolysis (pepsin) of *Comadia redtenbacheri* extracts and RWPTH: sequential hydrolysis (pepsin-trypsin) of *Comadia redtenbacheri* extracts. * Commercial inhibitor (NT: no treatment). Different uppercase letters (A–D) indicate statistically significant differences (p≤0.05) among treatments within *A. hesperiaris* samples and Enalapril, while lowercase letters (a–d) denote differences within *C. redtenbacheri* and Enalapril.

## Data Availability

No new data were created or analyzed in this study. Data sharing is not applicable to this article.

## Abbreviations

The following abbreviations are used in this manuscript:

ABTS	2,2′-azino-bis(3-ethylbenzothiazoline-6-sulfonic acid)
DPPH	2,2-diphenyl-1-picrylhydrazyl
ACE	Angiotensin Converting Enzyme
IC_50_	Inhibitory concentration at 50%
WW	*Aegiale hesperiaris* (maguey white worm)
RW	*Comadia redtenbacheri* (maguey red worm)
PH	Single hydrolysis with Pepsin only
PTH	Sequential Hydrolysis with Pepsin and Trypsin
WWPC	WW protein concentrate
RWPC	RW protein concentrate
SDS-PAGE	Sodium dodecyl sulfate polyacrylamide gel electrophoresis
AOAC	Association of Official Analytical Chemists
NFE	Nitrogen Free Extract
Tris-HCl	Tris (hydroxymethyl) aminomethane-hydrogen chloride buffer
BAEE	Benzyloxycarbonyl acid ethyl ester
kDa	Kilodaltons
B_1_	Positive control without inhibition
B_2_	Reagent blank
